# 艾曲泊帕治疗原发免疫性血小板减少症疗效及安全性的真实世界单中心数据分析

**DOI:** 10.3760/cma.j.cn121090-20231108-00257

**Published:** 2024-03

**Authors:** 喜凤 董, 亚兰 李, 念斌 李, 纬楠 林, 婷 王, 化泉 王, 丽娟 李, 文 瞿, 莉民 邢, 鸿 刘, 玉红 吴, 国锦 王, 嘉 宋, 晶 关, 晓明 王, 宗鸿 邵, 蓉 付

**Affiliations:** 1 天津医科大学总医院血液科，天津 300052 Department of Hematology, Tianjin Medical University General Hospital, Tianjin 300052, China; 2 天津市骨髓衰竭及癌性造血克隆防治重点实验室，天津 300052 Tianjin Key Laboratory of Bone Marrow Failure and Malignant Hemopoietic Clone Control, Tianjin 300052, China

**Keywords:** 艾曲泊帕, 原发免疫性血小板减少症, 有效性, 安全性, Eltrombopag, Primary immune thrombocytopenia, Efficacy, Safety

## Abstract

**目的:**

观察艾曲泊帕治疗原发免疫性血小板减少症（ITP）的疗效及安全性，并分析相关影响因素。

**方法:**

对自2018年1月至2022年3月在天津医科大学总医院血液科应用艾曲泊帕的198例ITP患者进行回顾性分析。

**结果:**

198例ITP患者，男70例、女128例，中位年龄45（7～88）岁，新诊断ITP 130例（65.7％），持续性ITP 25例（12.6％），慢性ITP 43例（21.7％）。基线出血评分：4分以下患者占84.3％，4分及以上患者占15.7％。艾曲泊帕6周内有效率（初始有效率）为78.8％，其中完全反应（CR）率为49.1％，CR1率为14.6％，CR2率为15.2％；中位起效时间7（7，14）d。艾曲泊帕25、50、75 mg剂量组初始反应率分别为74.1％、85.9％和60.0％（*P*＝0.031），50 mg剂量组初始反应率高于25 mg和75 mg剂量组。在治疗过程中，有70.7％的患者维持初始剂量，8.6％的患者调整到50 mg剂量组，6.1％的患者调整到75 mg剂量组，2例患者调整到100 mg剂量组。且调整剂量后25、50、75 mg各个剂量组的持续有效率分别为83.6％、85.3％和85.7％，差异无统计学意义；调整后，100 mg剂量组共4例，持续有效率为100.0％。艾曲泊帕治疗6周后，ITP患者出血评分总体降低，4分以上病例数降低为0，4分患者比例降低，1～2分患者比例变化不显著。艾曲泊帕治疗中，肝功能异常发生率为7.7％，经药物治疗均能好转，未见有合并血栓病例发生。ITP类型与巨核细胞数量分别是艾曲泊帕疗效的影响因素。新诊断的、持续性、慢性ITP患者对艾曲泊帕的初始有效率分别为85.3％、56％和76.2％（*P*＝0.003）；巨核细胞数量减少、正常和增多的ITP患者对艾曲泊帕的初始有效率分别为61.8％、87.1％和84.3％（*P*＝0.009）。

**结论:**

艾曲泊帕作为二线及以上治疗对成人ITP起效时间较快且具有良好的安全性。艾曲泊帕50 mg、25 mg剂量组相比较，50 mg剂量组初始有效率显著增高。新诊断类型的ITP、巨核细胞数量正常或增多的ITP患者对艾曲泊帕初始反应率更高。

## Abstract (en)

**Objective:**

This study aimed at investigating the efficacy and safety of eltrombopag in the treatment of adult primary immune thrombocytopenia（ITP）and evaluated the factors influencing its efficacy and side effects.

**Methods:**

A total of 198 patients with adult ITP who were admitted to Tianjin Medical University General Hospital between January 2018 and March 2022 were retrospectively analyzed. The efficacy of each starting dose of eltrombopag was evaluated, and adverse events were analyzed. The factors influencing efficacy were investigated, including sex, age, adult ITP type, platelet antibodies, and combined drug treatments.

**Results:**

Of the 198 patients, 70 males and 128 females with a median age of 45 years（18−88 years）were included; 130（65.7%）had newly diagnosed adult ITP, 25（12.6%）had persistent adult ITP, and 43（21.7%）had chronic adult ITP. The bleeding event scores at baseline were assessed; 84.3% had scores of <4 and 15.7% had scores of ≥4. The eltrombopag response rate（initial response）at 6 weeks was 78.8%（complete response [CR]: 49.0%; CR1: 14.6%; CR2: 15.2%）. The median response time to eltrombopag was 7（7, 14）days. The initial response rates to 25, 50, and 75 mg eltrombopag were 74.1%, 85.9%, and 60.0%, respectively（*P*＝0.031）. The initial response rate to the 50 mg dose was significantly higher than that of the 25-mg and 75-mg doses. Two patients received 100 mg as the starting dose, and their initial response was 0. Regarding dose adjustment, 70.7% of the patients remained on the starting dose, 8.6% underwent dose adjustment to 50 mg, and 6.1% underwent dose adjustment to 75 mg. Another two patients underwent dose adjustment to 100 mg. After dose adjustment, the persistent response rates were 83.6%, 85.3%, and 85.7% for the 25-, 50-, and 75-mg doses, respectively, with no significant difference. After dose adjustment, the sustained efficacy rate for the 100-mg dose（4 patients）was 100.0%. After 6 weeks of treatment with eltrombopag, the overall bleeding score of patients with ITP decreased. The number of patients with a score of ≥4 decreased to 0, the number of patients with a score of <4 decreased, and there was no significant change in the number of patients with a score of 1−2. The most common adverse event associated with eltrombopag was impaired liver function（7.7%）. No thrombosis events or other adverse events were observed. ITP type and number of megakaryocytes significantly affected the initial response to eltrombopag. The initial response rates to eltrombopag for newly diagnosed adult ITP, persistent adult ITP, and chronic adult ITP were 85.3%, 56.0%, and 76.2%, respectively（*P*＝0.003）. For megakaryocytes, the initial response rates were 61.8%, 87.1%, and 84.3%（*P*＝0.009）for the decreased, normal, and increased megakaryocyte groups, respectively.

**Conclusion:**

Eltrombopag, as a second-line or higher treatment for adult ITP, has a rapid onset of action and good safety. The initial response rate is significantly higher with a dose of 50 mg than with a dose of 25 mg. Patients with newly diagnosed ITP and those with normal or increased megakaryocyte numbers have a higher initial response rate to eltrombopag.

原发免疫性血小板减少症（ITP）是一种获得性自身免疫性出血性疾病[1]。目前成人ITP的一线治疗仍是糖皮质激素和丙种球蛋白，但在糖皮质激素减量或停药的过程中大部分患者出现复发，对于糖皮质激素不能耐受或者复发的患者，应启动二线治疗[2]。目前ITP的二线治疗包括TPO受体激动剂、利妥昔单抗和脾切除术[3]。艾曲泊帕是TPO受体激动剂，于2018年1月4日在中国获批上市，适用于既往对糖皮质激素、免疫球蛋白等治疗反应不佳的成人（≥18周岁）慢性ITP患者。此外，艾曲泊帕也用于因血小板减少和临床条件导致出血风险增加的血小板减少症患者。国内外已有多项临床试验提供其治疗ITP的疗效及安全性的相关数据[4]–[5]，但在实际的临床诊疗的过程中，ITP患者病情相对复杂，与临床试验纳入的ITP患者有入排标准的控制有所不同。故在真实世界的临床诊疗中，艾曲泊帕作为治疗ITP的二线药物，临床医师更加需要其疗效和安全性等数据。本文对天津医科大学总医院血液科自2018年1月以来应用艾曲泊帕作为二线及以上线数治疗的ITP患者病例资料进行回顾性分析，时间跨度近4年，总样本量198例，总结了临床应用艾曲泊帕治疗ITP的真实世界的数据，旨在为临床治疗ITP提供可信赖的循证医学数据。

## 病例与方法

一、研究对象

纳入2018年1月至2022年3月，在我科作为二线及以上药物使用艾曲泊帕治疗的198例ITP患者。诊断符合《成人原发免疫性血小板减少症诊断与治疗中国指南（2020年版）》标准[3]：包括病史及体检，至少连续2次血常规PLT减少，外周血涂片镜检血细胞形态无明显异常；脾脏一般不大；骨髓检查中常表现为巨核细胞增多或正常。排除继发性血小板减少症，包括自身免疫性疾病、甲状腺疾病、淋巴系统增殖性疾病等情况。

所有患者均依据病程长短分为：新诊断的ITP（确诊后3个月以内的患者）；持续性ITP（确诊后3～12个月血小板持续减少的患者）；慢性ITP（血小板持续减少超过12个月的患者）。

疗效标准[3]–[4]：完全反应（CR）：治疗后PLT≥100×10^9^/L且无出血表现。有效（R）：治疗后PLT≥30×10^9^/L，比基础PLT增加至少2倍，且无出血表现。此部分再细分为有效1（CR1）和有效2（CR2）：PLT（30～50）×10^9^/L为CR1，PLT（50～100）×10^9^/L为CR2。无效（NR）：治疗后PLT<30×10^9^/L，或PLT增加不到基础值的2倍，或有出血。复发：治疗有效后，PLT降至30×10^9^/L以下，或降至不到基础值的2倍，或出现出血症状。持续有效：患者疗效维持至开始治疗后6个月及以上。巨核细胞数目减少指巨核细胞数量小于7个；巨核细胞数目正常指7～35个；巨核细胞数目增多指大于35个。

二、数据采集

1. 统计研究对象的一般特征：包括年龄、性别、ITP诊断类型、出血评分[3]等。

2. 疾病特征及相关数据收集：明确诊断的ITP患者，经过一线治疗无效者，符合二线及以上治疗指标，启动艾曲泊帕治疗。本中心的患者，分析既往治疗情况，应用艾曲泊帕作二线治疗的例数为157例，三线治疗的例数为35例，四线治疗的例数为3例。按照艾曲泊帕起始剂量，分别为25、50、75、100 mg/d（仅有2例重症难治性ITP采取此剂量）组。PLT监测频率：开始治疗后最初6周，每周1～2次，6周之后每周1次，每周监测肝功能指标并根据肝功能情况及时调整治疗。

3. 药物调整剂量的相关数据采集：艾曲泊帕剂量调整原则：连续两周PLT<50×10^9^/L者，剂量增加25 mg/d，大部分患者最大剂量75 mg/d（50、75 mg/d依次递增），少数难治性ITP可加量至100 mg/d；PLT（150～250）×10^9^/L者，将剂量降至相邻的较低剂量（如50 mg/d减至25 mg/d）或降低给药频率（如25 mg/d减至25 mg隔日1次）；PLT>250×10^9^/L者暂停服用药物，待PLT<100×10^9^/L后继续用药，剂量为其停药前剂量，后期根据PLT下降水平再对剂量做调整。

4. 疗效和安全性指标：根据PLT水平，记录起效时间，判断出血评分的变化。分析年龄、性别、ITP诊断类型、血小板抗体、巨核细胞数量、其他自身抗体数目、既往用药情况与疗效进行相关性分析；记录治疗期间不良事件发生情况。

三、统计学处理

采用SPSS 20.0进行统计学分析。分类变量以例数（构成比）进行描述，采用Fisher精确概率法进行组间比较。双侧*P*<0.05为差异有统计学意义。

## 结果

一、ITP患者基线临床特征

本中心近4年来应用艾曲泊帕治疗的198例ITP患者中，男70例、女128例，中位年龄45（17～88）岁。新诊断ITP 130例（65.7％），持续性ITP 25例（12.6％），慢性ITP 43例（21.7％）。基线出血评分：4分以下患者占84.3％，4分及以上患者占15.7％，有伴随用药的患者占76.8％。本临床研究中纳入的患者一线治疗均为静脉丙种球蛋白或糖皮质激素，部分患者曾应用过环孢素A、利妥昔单抗、环磷酰胺、TPO等药物作为二线治疗，故部分患者选用艾曲泊帕治疗时为三线或四线治疗。198例患者中，合并高血压、糖尿病和冠心病等慢性病比例高，患有以上3种常见病中的至少2种者为27例，单患有高血压21例、冠心病10例、糖尿病7例。2例患者有肝硬化，1例为丙肝携带者，无活动性乙肝患者，1例有癫痫病史；此外1例患者合并重症肌无力，1例合并白癜风。

二、疗效分析

艾曲泊帕治疗6周内的初始有效率为78.8％，其中CR率为49.0％，CR1率为14.6％，CR2率为15.2％，持续有效率为71.7％，其中CR率为41.9％，CR1率为15.2％，CR2率为14.6％。艾曲泊帕不同起始剂量组的初始反应率和调整剂量后的持续有效率见表1。3个剂量组中，50 mg剂量组初始反应率高于25 mg和75 mg剂量组（Fisher，*P*＝0.031）。在治疗过程中，有70.7％的患者维持初始剂量，8.6％的患者调整到50 mg剂量组，6.1％的患者调整到75 mg剂量组，2例患者调整到100 mg剂量组。且调整剂量后各个剂量组的持续有效率相近，差异无统计学意义。6周后患者的出血评分评价见表2。我们可以看出，经艾曲泊帕治疗后，ITP患者出血评分总体降低，4分以上病例数降低为0，评分为0分患者比例增加，4分患者比例显著降低，而分布于1～2分患者比例变化不显著。154例艾曲泊帕起效时间在7（7，14）d。有2例患者因不耐受而停药。肝功能异常发生率7.7％，经治疗后均好转，未见血栓性事件及其他不良事件发生。

**表1:** 艾曲泊帕治疗原发免疫性血小板减少症起始与调整的剂量分组及其疗效^a^［例数（％）］

剂量	初始例数	初始有效	调整后例数	可持续随访例数	持续有效
25 mg	85	63（74.1）	81	66	56（83.6）
50 mg	99	85（85.9）	88	75	64（85.3）
75 mg	10	6（60.0）	18	16	14（85.7）
100 mg	2	0	4	4	4（100.0）

*P*值		0.031			0.944

注 a：2例患者因不耐受而停药，未纳入分析，调整剂量后例数为191，其中可获得疗效数据的161例

**表2:** 198例原发免疫性血小板减少症患者基线及艾曲泊帕治疗6周出血评分［例数（％）］

出血评分	基线	艾曲泊帕治疗6周
0	28（14.1）	95（48.0）
1	65（32.8）	66（33.3）
2	44（22.2）	34（17.2）
3	30（15.2）	2（15.1）
4	11（5.6）	1（0.5）
5	10（5.1）	0
6	4（2.0）	0
7	6（3.0）	0

三、影响疗效的相关因素分析

单因素分析性别、年龄、血小板抗体、合并其他自身免疫性抗体种类对初始有效率有无显著影响。结果见表3，不同ITP类型（新诊断、持续性和慢性ITP）艾曲泊帕的初始有效率分别为85.3％、56.0％和76.2％（*P*＝0.003）；巨核细胞数量分为减少、正常和增多，初始有效率分别为61.8％、87.1％和84.3％（*P*＝0.009）。而性别、年龄、血小板抗体、合并其他自身免疫性抗体种类对艾曲泊帕治疗ITP的初始有效率无显著影响。

**表3:** 影响艾曲泊帕治疗原发免疫性血小板减少症（ITP）疗效的单因素分析［例数（％）］

因素	有效	*P*值
ITP类型		0.003
新诊断	110（85.3）	
持续性	13（56.0）	
慢性	32（76.2）	
巨核细胞数量		0.009
减少	21（61.8）	
正常	27（87.1）	
增多	91（84.3）	
年龄		1.000
>40岁	91（79.8）	
≤40岁	65（79.3）	
性别		0.557
男性	57（82.6）	
女性	99（78.0）	
血小板抗体		0.568
阴性	97（77.0）	
阳性	18（85.7）	
合并其他自身免疫性抗体种类		0.450
0种	66（83.5）	
1～2种	62（75.6）	
3～6种	13（82.2）	
曾用药物种类		0.691
≤2种	135（84.0）	
≥3种	21（75.0）	

## 讨论

ITP虽然是一种良性疾病，但目前仍有部分患者对一线治疗效果不佳，或者是对糖皮质激素治疗依赖，严重影响患者生存和生活质量。自艾曲泊帕问世以来，国内外开展了多项关于其治疗儿童或成人ITP的临床试验并获得了令人满意的结果。总体来看，艾曲泊帕可迅速提升ITP患者的PLT、改善临床出血症状，总体治疗有效率可达50％～80％[6]–[8]。而且，长期用药的患者中有三分之一左右可在停药后进入持续缓解状态。基于国内外大型临床试验的数据支持，欧洲药物管理局及美国FDA已批准了艾曲泊帕用于治疗对糖皮质激素、丙种球蛋白反应不佳的成人及儿童慢性ITP患者。且艾曲泊帕在中国已获批治疗ITP的适应证（2016版原发免疫性血小板减少症诊治的中国共识推荐促血小板生成药物为ITP治疗的二线治疗药物之一）[9]。艾曲泊帕作为第二代促血小板生成药物，是一种口服、合成非肽类小分子药物，其作用位点是TPO受体的跨膜区域，可诱导造血干细胞向巨核细胞增殖、分化、多谱系造血恢复的作用[10]，其在体内的主要代谢器官为肝脏，血浆清除半衰期为26～35 h。由于与TPO受体作用的位点不同，艾曲泊帕与内源性TPO之间并不存在交叉反应及竞争抑制[11]–[12]。文献报道的艾曲泊帕的毒性反应有骨髓纤维化、血栓形成、肝毒性、反弹性血小板减少及血液恶性肿瘤等[13]–[15]。本文分析的安全性数据方面，艾曲泊帕虽然存在肝功能的损伤，但发生率不高，肝功能异常发生率7.7％，除了1例患者因此不良反应而停药外，其余合并肝损伤患者均经治疗后好转，且本中心的198例患者中均未见血栓性事件及其他不良事件发生。

目前推荐艾曲泊帕治疗ITP的给药方案有两种：连续用药方案：推荐25 mg/d或50 mg/d为起始剂量治疗2周，若患者PLT仍<50×10^9^/L，应适当加大药物用量，最高可达75 mg/d；如果患者此时PLT为（200～400）×10^9^/L，应适当降低药量，最低为12.5 mg/d；75 mg/d艾曲泊帕连续治疗4周仍无反应者判定药物治疗失败，建议停药。选择性间歇给药方案：给药剂量为50 mg/d或75 mg/d（东亚裔患者为25 mg/d），持续治疗2周以上，假如患者PLT超过200×10^9^/L，降低用药频率为每周5次，2周后再次评估血小板水平，若能够继续维持在100×10^9^/L以上的水平，再次降低用药频率；当出现患者PLT<20×10^9^/L的情况时，则要恢复至上一阶段给药频率。此外，若患者在每周一次的用药频率下，多达2周以上的时间PLT>100×10^9^/L，可尝试停药[16]–[17]。

本中心采用的为第一种方式的给药方案。本文为本血液中心近4年临床应用艾曲泊帕治疗ITP的真实世界的数据总结，旨在为临床治疗ITP提供可信赖的循证医学数据。本中心数统计结果显示：艾曲泊帕治疗6周内的初始有效率78.8％，且艾曲泊帕不同起始剂量组的初始反应率分别为：25 mg剂量组为74.1％；50 mg剂量组为85.9％；75 mg剂量组为60.0％，差异有统计学意义。故建议起始用量为50 mg/d，早期反应率（6周内）优于25 mg/d，小部分患者75mg起始量，有效率理想，未见严重不良反应，监测肝功能。我们将此结果与舒华娥等对重庆开州人民医院17例艾曲泊帕治疗ITP的临床分析、苏璟等对联勤保障部队第九六七医院血液科75例患者的临床分析等结果进行比较，以上中心均未将艾曲泊帕不同起始剂量治疗ITP的疗效进行分层比较[18]–[19]。另外黄月婷等[20]75例ITP的临床分析，对不同起始剂量的艾曲泊帕治疗ITP的疗效进行了分析，得出25 mg剂量组有效率偏低，建议50 mg作为起始剂量的结论。此结论虽与本中心临床研究结论相同，但是其25 mg剂量组有效率22.22％，低于本中心25 mg剂量组74.1％的数据。分析原因可能为本中心数据与其所在单位收治患者构成比不同。黄月婷等[20]文章中强调其70多例患者中，以慢性ITP患者为主，但有13例经一线治疗无效或病情反复的新诊断及持续性ITP。而本中心收治患者中新诊断的患者比例高达60％以上，可能这是对25 mg剂量组对艾曲泊帕反应率相对高的原因之一。目前我们正在进行对于新诊断的ITP应用艾曲泊帕治疗的相关临床试验，能更好地回答艾曲泊帕起始剂量疗效的问题。另外本中心数据中，在198例患者中，有2例患者初始剂量为100 mg，均为重症；另有2例患者逐渐加量至100 mg，这两例均为80岁以上高龄的慢性ITP患者，合并症多，对激素及免疫调节治疗反应差，出血倾向严重，应用艾曲泊帕100 mg剂量后远期效果好，能达到脱离输注血小板，且PLT达到30×10^9^以上的安全水平，降低了出血风险并改善其相关症状。

进一步按照患者年龄、性别、诊断类型、血小板抗体等等诸多因素分析其对艾曲泊帕疗效的影响，结果提示：相比较持续性和慢性ITP，新诊断类型的ITP对艾曲泊帕治疗反应率更高，可达85.3％。巨核细胞数量对反应率有影响。巨核细胞数量正常或增多反应率较好，有效率可达80％以上，而巨核细胞减少患者，有效率仅为61.8％，差异有统计学意义。此外患者年龄对于初始反应率的影响差异无统计学意义。性别、血小板抗体是否阳性对于艾曲泊帕治疗ITP的初始反应率差异无统计学意义。分析合并的自身免疫性抗体的情况：其中66例的患者未合并自身抗体。62例患者合并1～2种自身免疫性抗体，这些抗体的主要以抗核抗体为主，部分患者在抗核抗体的基础上合并RO-52或者其他风湿免疫抗体阳性。仅仅有13例患者合并3种及以上自身免疫抗体，此部分患者在临床诊疗过程中均已经请过风湿免疫科专家会诊，不能明确诊断为结缔组织疾病。且合并风湿免疫抗体3个及以上的患者人数有限，不能进行进一步分层分析。但是本中心的数据表明，这些不同程度合并自身免疫性抗体的ITP患者，虽然合并自身免疫抗体越少的ITP患者，对艾曲泊帕反应率越好，但差异无统计学意义。发生率最高的不良事件为肝损害（转氨酶、胆红素增高），均自行好转或经停药、对症治疗后恢复。

本中心数据表明，艾曲泊帕治疗成人ITP具有起效快、有效率高的优点，用药方便，耐受性好。对于长期血小板减少、一线药物治疗无效、复发或不能耐受糖皮质激素的患者，艾曲泊帕均可作为二线治疗选择。
